# Two-Year clinical outcomes after coronary bifurcation stenting in older patients from Korea and Italy

**DOI:** 10.3389/fcvm.2023.1106594

**Published:** 2023-03-23

**Authors:** Ju Hyeon Kim, Luca Franchin, Soon Jun Hong, Jung-Joon Cha, Subin Lim, Hyung Joon Joo, Jae Hyoung Park, Cheol Woong Yu, Do-Sun Lim, Ovidio De Filippo, Hyeon-Cheol Gwon, Francesco Piroli, Hyo-Soo Kim, Wojciech Wanha, Ki Hong Choi, Young Bin Song, Giuseppe Patti, Chang-Wook Nam, Francesco Bruno, Jeehoon Kang, Pier Paolo Bocchino, Gaetano Maria De Ferrari, Bon-Kwon Koo, Fabrizio D’Ascenzo

**Affiliations:** ^1^ Department of Cardiology, Cardiovascular Center, Korea University Anam Hospital, Korea University College of Medicine, Seoul, Republic of Korea; ^2^ Division of Cardiology, Cardiovascular and Thoracic Department, University of Turin, Turin, Italy; ^3^ Division of Cardiology, Department of Medicine, Heart Vascular Stroke Institute, Samsung Medical Center, Sungkyunkwan University School of Medicine, Seoul, Republic of Korea; ^4^ Department of Internal Medicine and Cardiovascular Center, Seoul National University College of Medicine, Seoul, Republic of Korea; ^5^ Department of Cardiology and Structural Heart Diseases, Medical University of Silesia, Katowice, Poland; ^6^ Department of Thoracic and Cardiovascular Diseases, Maggiore Della Carita Hospital, Novara, Italy; ^7^ Department of Internal Medicine, Keimyung University Dongsan Medical Center, Daegu, Republic of Korea

**Keywords:** bifurcation, coronary intervention, left main coronary artery (LMCA) disease, old age, elderly

## Abstract

**Background:**

Older patients who treated by percutaneous coronary intervention (PCI) are at a higher risk of adverse cardiac outcomes. We sought to investigate the clinical impact of bifurcation PCI in older patients from Korea and Italy.

**Methods:**

We selected 5,537 patients who underwent bifurcation PCI from the BIFURCAT (comBined Insights from the Unified RAIN and COBIS bifurcAtion regisTries) database. The primary outcome was a composite of target vessel myocardial infarction, clinically driven target lesion revascularization, and stent thrombosis at two years.

**Results:**

In patients aged ≥75 years, the mean age was 80.1 ± 4.0 years, 65.2% were men, and 33.7% had diabetes. Older patients more frequently presented with chronic kidney disease (CKD), severe coronary calcification, and left main coronary artery disease (LMCA). During a median follow-up of 2.1 years, older patients showed similar adverse clinical outcomes compared to younger patients (the primary outcome, 5.7% vs. 4.5%; *p *= 0.21). Advanced age was not an independent predictor of the primary outcome (*p *= 0.93) in overall patients. Both CKD and LMCA were independent predictors regardless of age group.

**Conclusions:**

Older patients (≥75 years) showed similar clinical outcomes to those of younger patients after bifurcation PCI. Advanced age alone should not deter physicians from performing complex PCIs for bifurcation disease.

## 1. Introduction

As the number of older patients with coronary artery disease (CAD) increases, clinicians face more complex and severe disease, such as true coronary bifurcation lesions, with worse clinical outcomes (1). Comorbidities are more prevalent in older patients with CAD (2, 3), and they show a higher risk of adverse clinical events as well as periprocedural complications compared to younger patients (4). Moreover, frailty, cognitive impairment, and functional disability, as well as advanced age, have been linked to increased cardiovascular and all-cause mortality (5–7). Consequently, older patients have been undertreated with coronary revascularization compared to their younger counterparts (8–10) and thus underrepresented in clinical trials using percutaneous coronary intervention (PCI) (8, 11). In real-world clinical practice, physicians opt for conservative treatment in older patients, although the guidelines do not suggest any age limitations in the use of PCI (12).

Numerous studies have evaluated the clinical outcomes of complex bifurcation stenting (13–15), and PCI for bifurcation CAD remains challenging and requires complex procedures. Furthermore, there is a limited data about the prognosis of older patients with bifurcation CAD. Therefore, we aimed to evaluate the impact of advanced age on clinical outcomes in a large multinational cohort that underwent bifurcation PCI.

## 2. Methods

### 2.1. Source of data

The comBined Insights From the Unified RAIN and COBIS bifurcAtion regisTries (BIFURCAT) database was created by merging two large datasets (16). The COBIS III (COronary BIfurcation Stenting [COBIS] III, NCT03068494) registry is a multicenter, nationwide cohort in South Korea that includes consecutive patients who underwent PCI for bifurcation CAD with second-generation drug-eluting stents (DESs) from January 2010 to December 2014 (17). The retrospective RAIN (veRy Thin Stents for Patients With Left mAIn or bifurcatioN in Real Life [RAIN], NCT03544294) registry included consecutive patients whose coronary bifurcations and/or unprotected left main coronary artery disease (LMCA) were treated with very thin DESs (<100 mm) in 15 European centers from June 2015 to December 2017 (18). The detailed information for both registries has been previously reported.

PCI was performed as per the current practice guidelines by the Korean Society of Interventional Cardiology and the European Society of Cardiology (12). Periprocedural anticoagulation was performed using either low-molecular-weight or unfractionated heparin to maintain an activated clotting time of 250–300 s during PCI. All patients received aspirin (300 mg) and P2Y12 inhibitor loading doses (300 or 600 mg for clopidogrel, 180 mg for ticagrelor, and 60 mg for prasugrel) before PCI, unless they had previously received these antiplatelet medications. The stenting strategy (provisional or upfront two-stent technique), access sites, type of implanted DES, and use of intracoronary imaging were left to the discretion of the operator. All patients were prescribed standard maintenance doses of dual antiplatelet drugs after bifurcation PCI. The duration of dual antiplatelet drugs was left to the decisions of the caring physicians.

### 2.2. Study population

A total of 5,537 patients from Korea and Italy were included in the BIFURCAT registry (Supplementary Figure S1). A detailed description of the study population, including the demographic, angiographic, and procedural data, was obtained using a web-based system. Additional data, such as the duration of prescribed antiplatelet therapy, was obtained from a review of electronic health records or telephone contact if necessary. All clinical outcomes from the participating centers were reviewed and validated by an independent clinical event-adjudicating committee.

### 2.3. Study variables and outcomes

The clinical characteristics of older patients (aged ≥75 years) were compared to those of younger patients (aged <75 years). The primary outcome was a composite of target vessel myocardial infarction (MI), clinically driven target lesion revascularization (TLR), and stent thrombosis (ST) at two years post-PCI. All-cause death and any MI were secondary outcomes, as were the single components of the primary outcome. MI was defined as an elevation of the cardiac enzyme level greater than the upper limit of normal with ischemic symptoms or signs indicative of ischemia, not related to the index PCI (19). TLR was considered as a repeat PCI of the lesion within 5 mm of the inserted stent. ST was classified as definite, probable, or possible using the definitions of the Academic Research Consortium (20). Both definite and probable cases were included as STs in this analysis. Outcomes of interest were appraised according to age at the time of the index PCI (aged ≥75 vs. <75 years).

### 2.4. Statistical analysis

Continuous variables were reported as means ± standard deviations, whereas categorical variables were reported as counts (percentages). Group comparisons were performed using parametric (unpaired *t*-test) and nonparametric (Mann–Whitney *U* test) tests for continuous variables, whereas the chi-square test was used for categorical variables. The cumulative incidence of clinical outcome events was calculated based on Kaplan–Meier censoring estimates, and intergroup comparisons were assessed using the log-rank test. The relationships between age and clinical outcomes were explored using restricted cubic splines (21). A multivariable Cox model was used to calculate adjusted hazard ratios (HRs) and 95% confidence intervals (CIs). Variables with significance in the univariable analysis (*p *< 0.2) or clinically relevant variables were included in the final model to determine independent predictors of the primary outcome. The final model included age, hypertension, diabetes mellitus, chronic kidney disease (CKD), prior history of PCI and/or coronary artery bypass graft surgery, left ventricular (LV) ejection fraction <40%, presentation of acute coronary syndrome (ACS), LMCA, true bifurcation lesion, severe calcification, and stenting technique. True bifurcation lesions were defined as Medina (1,1,1; 1,0,1; and 0,1,1) lesions (22). To account for the effect of age, the sub-distribution HR was calculated using the Fine-Gray competing risk model (23). In addition, a propensity score (PS) matching analysis was also performed, and each patient in the older group was matched with those in the younger group at a 1:1 ratio using the nearest neighbor method, with a caliper width equal to 0.2 of the standard deviation of the logit PS. Statistical analyses were performed using R Statistical Software (version 4.1.2; R Foundation for Statistical Computing, Vienna, Austria). Except for the subgroup interaction analysis (*p *< 0.1), *p* values of 0.05 were considered statistically significant.

## 3. Results

### 3.1. Clinical characteristics

A total of 5,537 patients were included in the current analysis, and 1,415 patients (26%) were aged ≥75 years (the older group). The median age of all study patients was 67 (IQR, 58–75) years (Supplementary Figure S2). Table 1 presents the clinical characteristics, including baseline demographics. The mean age was 80.1 ± 4.0 years and 65.2% were men in older patients. Comorbidities, including hypertension (77.4% vs. 62.2%), CKD (23.7% vs. 9.3%), and history of MI (22.5% vs. 15.2%), were more frequent in older patients. Older patients presented more frequently with LMCA (33.1% vs. 28.6%, *p *= 0.001) and more complex coronary lesions, including a higher proportion of true bifurcation lesions (51.8% vs. 46.4%) and severe calcification (24.5% vs. 15.0%). Overall, PCI was performed with the two-stent strategy in 17.6% of patients, and older patients showed a shorter stent length of the main branch with a smaller diameter than younger patients.

**Table 1:** Comparison of clinical characteristics.

Characteristics	Overall (*n *= 5,537)	Age <75 (*n *= 4,122)	Age ≥75 (*n *= 1,415)	*P-*value
**Demographics**				
Age, years	66.2 ± 11.3	61.5 ± 8.9	80.1 ± 4.0	
Male	4,228 (76.4%)	3,305 (80.2%)	923 (65.2%)	<0.001
**Medical history**				
Hypertension	3,657 (66.0%)	2,562 (62.2%)	1,095 (77.4%)	<0.001
Hyperlipidemia	2,736 (49.4%)	2,026 (49.2%)	710 (50.2%)	0.525
Diabetes	1,834 (33.1%)	1,357 (32.9%)	477 (33.7%)	0.609
Current smoker	1,408 (25.4%)	1,274 (30.9%)	134 (9.5%)	<0.001
History of MI	944 (17.0%)	626 (15.2%)	318 (22.5%)	<0.001
History of CABG	157 (2.8%)	96 (2.3%)	61 (4.3%)	<0.001
History of PCI	1,244 (22.5%)	892 (21.6%)	352 (24.9%)	0.013
Chronic kidney disease	718 (13.0%)	383 (9.3%)	335 (23.7%)	<0.001
**Clinical presentation**				
LV ejection fraction, %	57.7 ± 9.5	58.3 ± 9.1	56.2 ± 10.5	<0.001
LV ejection fraction <40%	361 (6.5%)	214 (5.2%)	147 (10.4%)	<0.001
Acute coronary syndrome	3,231 (58.4%)	2,378 (57.7%)	853 (60.3%)	0.094
**Angiographic characteristics**				
LMCA	1,647 (29.7%)	1,178 (28.6%)	469 (33.1%)	0.001
Severe calcification	966 (17.4%)	619 (15.0%)	347 (24.5%)	<0.001
Diffuse lesion	2,045 (36.9%)	1,488 (36.1%)	557 (39.4%)	0.030
True bifurcation lesion	2,645 (47.8%)	1,912 (46.4%)	733 (51.8%)	<0.001
Medina classification				
1,1,1	1,780 (32.1%)	1,301 (31.6%)	479 (33.9%)	
1,0,1	436 (7.9%)	295 (7.2%)	141 (10.0%)	
0,1,1	429 (7.7%)	316 (7.7%)	113 (8.0%)	
1,1,0	1,366 (24.7%)	991 (24.0%)	375 (26.5%)	
1,0,0	544 (9.8%)	429 (10.4%)	115 (8.1%)	
0,1,0	751 (13.6%)	610 (14.8%)	141 (10.0%)	
0,0,1	231 (4.2%)	180 (4.4%)	51 (3.6%)	
**Procedural characteristics**				
Two-stent strategy	977 (17.6%)	711 (17.2%)	266 (18.8%)	0.201
Number of total stents	1.19 ± 0.4	1.18 ± 0.4	1.20 ± 0.4	0.068
MB, stent length (mm)	25.9 ± 11.8	26.3 ± 12.1	24.9 ± 10.9	<0.001
MB, stent length >30 mm	1,305 (23.6%)	994 (24.4%)	311 (22.1%)	0.089
MB, stent diameter (mm)	3.12 ± 0.6	3.13 ± 0.6	3.10 ± 0.6	0.064
MB, stent diameter <3.0 mm	1,586 (28.6%)	1,131 (27.4%)	455 (32.2%)	0.001

Values are presented as the mean ± standard deviation or n (%). CABG, coronary artery bypass graft surgery; LV, left ventricle; LMCA, left main coronary artery disease; MB, main branch; MI, myocardial infarction; PCI, percutaneous coronary intervention.

### 3.2. Incidence of clinical outcomes

**Figure 1:**
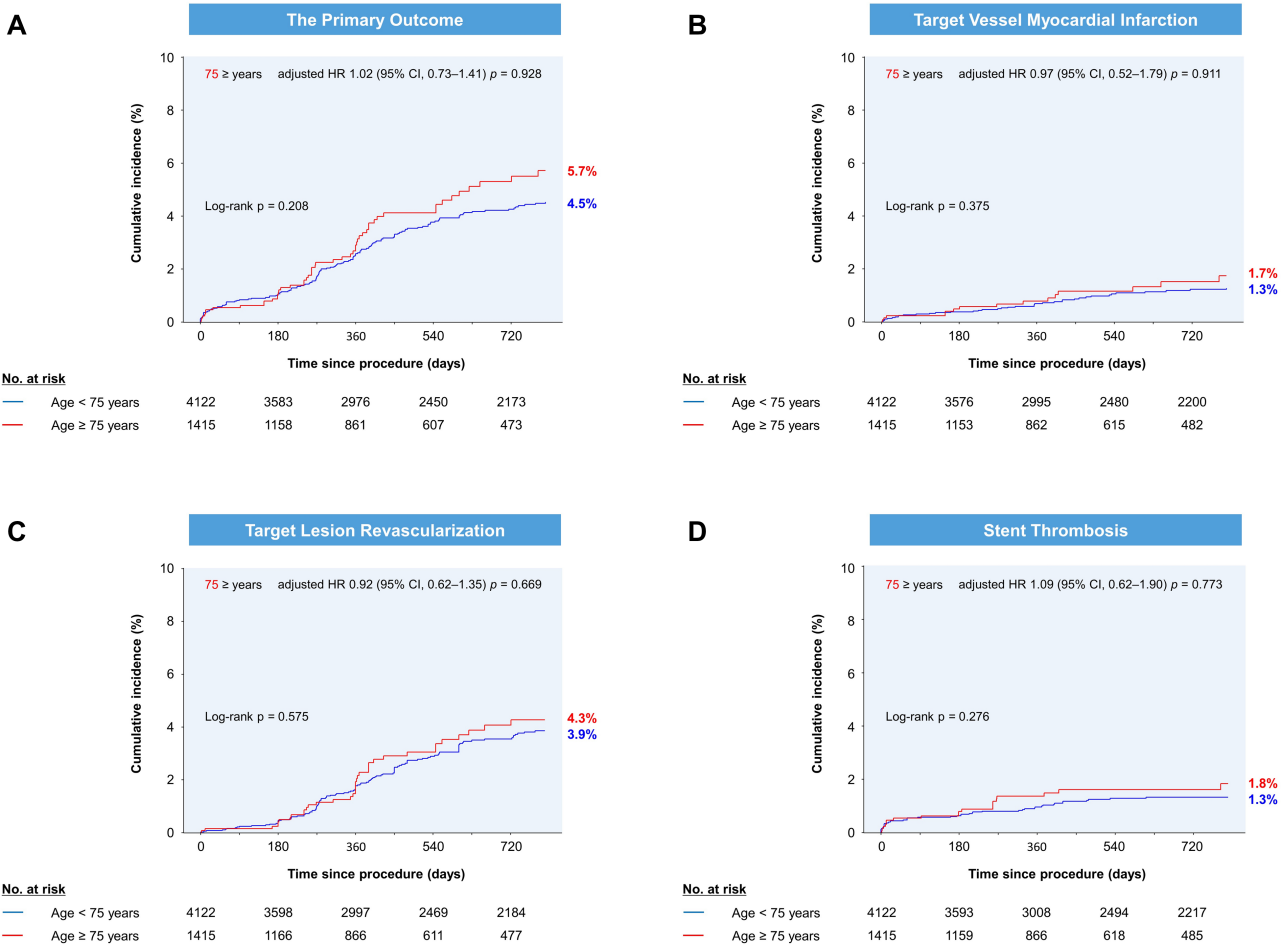
Cumulative incidence of (**A**) the primary outcome, (**B**) target vessel myocardial infarction, (**C**) target lesion revascularization, and (**D**) stent thrombosis by age group. The Kaplan-Meier estimates at 720 days are shown. HR, hazard ratio; CI, confidence interval.

**Figure 2:**
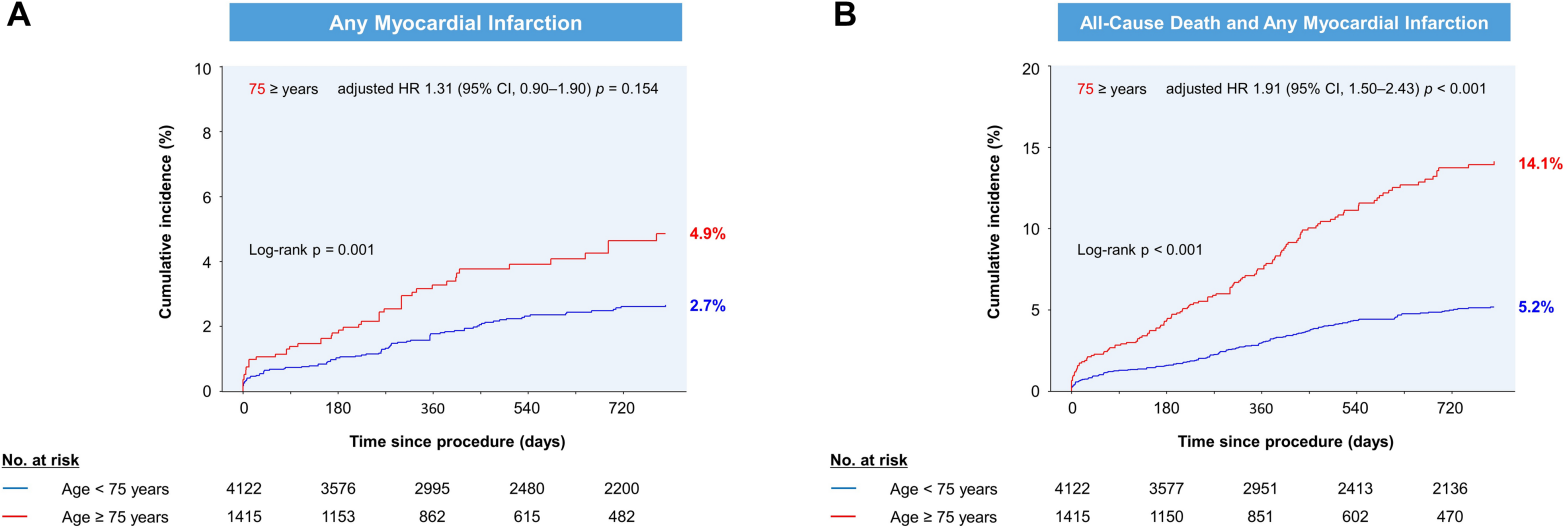
Cumulative incidence of (**A**) any myocardial infarction and (**B**) combined all-cause death and any myocardial infarction by age group. The Kaplan-Meier estimates at 720 days are shown. HR, hazard ratio; CI, confidence interval.

**Figure 3:**
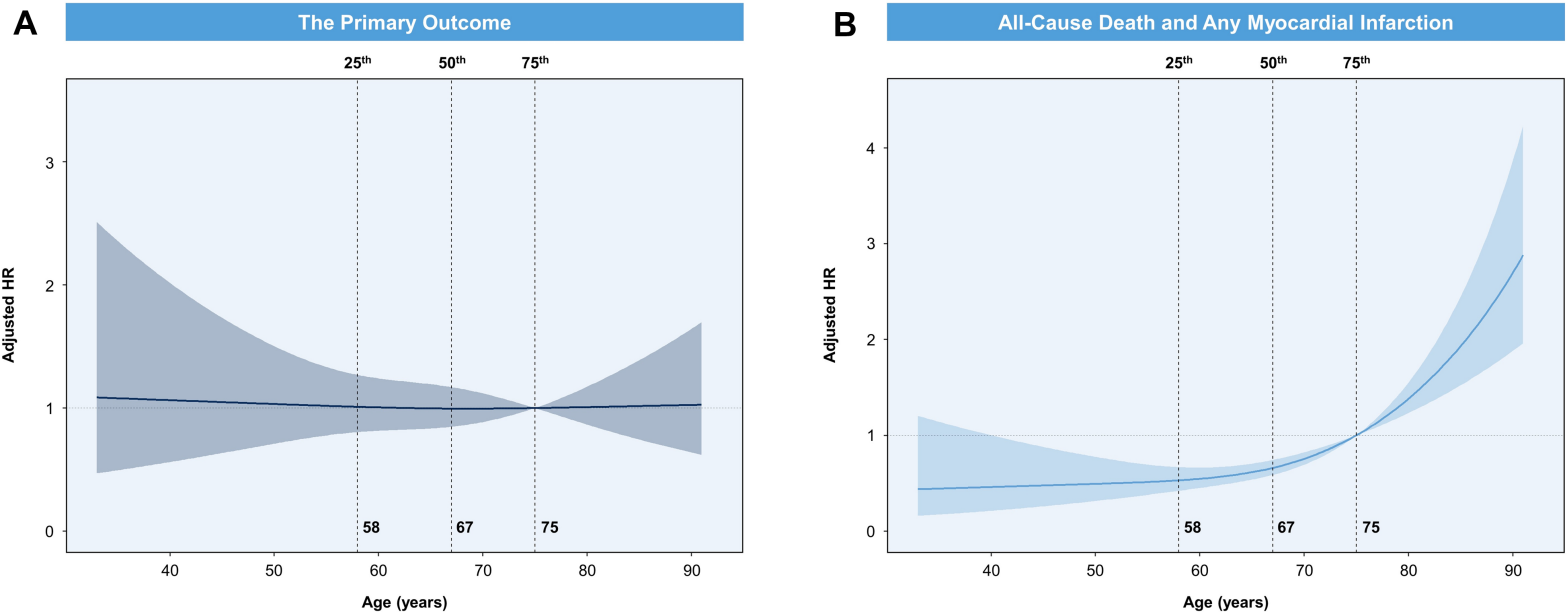
(**A**) spline curve for the association of age with the hazard of the primary outcome. (**B**) Spline curve for the association of age with the hazard of combined all-cause death and any myocardial infarction.

During a median follow-up of 2.1 (IQR, 0.9–2.2) years, the cumulative incidence of the primary outcome was similar between older and younger patients (*p *= 0.208; Figure 1). Older patients had similar rates of target vessel MI, clinically driven TLR, and ST compared to younger patients (Table 2). After adjustment, advanced age was not associated with the primary outcome (adjusted HR, 1.02; 95% CI, 0.73–1.41). In contrast, higher rates of any MI (4.9% vs. 2.7%) or combined all-cause death and any MI (14.1% vs. 5.2%) were observed in older patients (Figure 2). As a continuous variable, age had no effect on the adjusted risk of the primary outcome, and it had a curvilinear effect on the adjusted risk of combined all-cause death and any MI (Figure 3).

**Table 2:** Incidence and risk of the primary and secondary outcomes at 2 years.

Characteristics	Age <75 (*n *= 4,122)	Age ≥75 (*n *= 1,415)	Log-rank *P-*value	Adjusted HR* [95% CI]
**Primary outcome**	141 (4.5%)	51 (5.7%)	0.208	1.02 [0.73–1.41]
**Secondary outcomes**				
Target vessel MI	39 (1.3%)	15 (1.7%)	0.375	0.97 [0.52–1.79]
Target lesion revascularization	114 (3.9%)	36 (4.3%)	0.575	0.92 [0.62–1.35]
Stent thrombosis	45 (1.3%)	19 (1.8%)	0.276	1.09 [0.62–1.90]
Any MI	86 (2.7%)	48 (4.9%)	0.001	1.31 [0.90–1.90]
All-cause death + any MI	163 (5.2%)	136 (14.1%)	<0.001	1.91 [1.50–2.43]†

Values are presented as numbers (an estimate of the cumulative incidence of events over time). The primary outcome was defined as a composite of target vessel myocardial infarction, clinically driven target lesion revascularization, and stent thrombosis.

* Adjusted for hypertension, diabetes, chronic kidney disease, previous revascularization, left ventricular ejection fraction, presentation as acute coronary syndrome, left main disease, true bifurcation lesion, severe calcification, and the two-stent strategy.

†*p *< 0.001. CI, confidence interval; HR, hazard ratio; MI, myocardial infarction.

### 3.3. Predictors of the primary outcome

The independent predictors of the primary outcome were CKD, LMCA, and the two-stent strategy (Table 3). LMCA was the strongest predictor of the primary outcome (adjusted HR 2.07; 95% CI, 1.53–2.79). After adjustment with the Cox regression model, age ≥75 years was not an independent factor associated with the primary outcome. When all-cause death was modelled as a single competing outcome, the adjusted sub-distribution HR of the primary outcome was 0.98 (95% CI, 0.69–1.38; *p *= 0.91) for the older age group (Table 4). The multivariable-adjusted independent predictors of the primary outcome according to age subgroup are shown in Supplementary Table S1. In the older group, the presence of LMCA was a significant predictor (adjusted HR, 2.35; 95% CI, 1.31–4.21; *p *= 0.004), and CKD (adjusted HR, 1.85; 95% CI, 0.94–3.63; *p *= 0.073) showed a borderline significance. In the younger group, hypertension, diabetes, CKD, LV ejection fraction <40%, LMCA, and the two-stent strategy were important in predicting the risk of the primary outcome. In the subgroup analysis according to diabetes, advanced age (≥75 years) showed a significant interaction for the primary outcome (*p* for interaction = 0.036; Supplementary Figure S3).

**Table 3:** Predictors of the primary outcome in the overall cohort.

Variables	Univariable HR (95% CI)	*P-*value	Multivariable HR (95% CI)	*P-*value
Age ≥75 (years)	1.23 [0.89–1.69]	0.209	1.02 [0.73–1.41]	0.928
Hypertension	1.56 [1.14–2.15]	0.006	1.34 [0.97–1.87]	0.078
Diabetes	1.58 [1.19–2.11]	0.002	1.35 [1.00–1.81]	0.049
Chronic kidney disease	2.28 [1.59–3.29]	<0.001	1.95 [1.32–2.89]	<0.001
Previous PCI/CABG	1.29 [0.93–1.78]	0.127	1.02 [0.73–1.42]	0.919
LV ejection fraction <40%	1.73 [1.08–2.78]	0.023	1.54 [0.95–2.49]	0.082
Presentation as acute coronary syndrome	0.83 [0.62–1.10]	0.188	0.86 [0.64–1.14]	0.286
LMCA	2.20 [1.66–2.93]	<0.001	2.07 [1.53–2.79]	<0.001
True bifurcation lesion	1.36 [1.02–1.80]	0.036	1.20 [0.87–1.65]	0.266
Severe calcification	1.76 [0.97–3.20]	0.062	0.77 [0.54–1.12]	0.172
Two-stent strategy	2.46 [1.83–3.31]	<0.001	2.03 [1.45–2.84]	<0.001

CABG, coronary artery bypass graft surgery; CI, confidence interval; HR, hazard ratio; PCI, percutaneous coronary intervention; LV, left ventricle; LMCA, left main coronary artery disease.

**Table 4:** Crude and adjusted hazard ratios of the primary outcome at 2 years.

Age ≥75 (vs. Age < 75)	Crude HR (95% CI)	*P-*value	*Adjusted HR (95% CI)	*P-*value
Cox proportional hazard model	1.23 [0.89–1.69]	0.209	1.02 [0.73–1.41]	0.928
Competing risks†	1.18 [0.86–1.63]	0.320	0.98 [0.69–1.38]	0.910

* Adjusted for hypertension, diabetes, chronic kidney disease, previous revascularization, left ventricular ejection fraction, presentation as acute coronary syndrome, left main disease, true bifurcation lesion, severe calcification, and the two-stent strategy.

†All-cause death was modelled as a single competing outcome using the Fine-Gray method. CI, confidence interval; HR, hazard ratio.

### 3.4. Propensity score matching analysis

After 1: 1 PS matching, 2,634 patients were selected (the older group, *n* = 1,317). Demographic and clinical parameters at baseline were well balanced, and the standardized mean differences between the groups were <10.0% for all variables (Supplementary Table S2). The incidences of the primary outcome at 2 years were comparable between groups, and the rates of combined all-cause death and any MI were higher in older patients (Supplementary Table S3).

## 4. Discussion

The current study included 5,537 patients with bifurcation CAD and involved a real-world multinational registry created to evaluate the incidence and predictors of adverse cardiac events. The main findings of the present analysis are as follows: (1) the risk of the primary outcome was similar in older patients who underwent coronary bifurcation stenting compared to younger patients; (2) after adjustment, advanced age showed a significant association with an increased risk for combined all-cause death and any MI; and (3) both CKD and LMCA were significant predictors of the primary outcome regardless of age after bifurcation PCI.

Bifurcation CAD accounts for approximately 20% of all contemporary PCI and represents a challenging subgroup with poor clinical outcomes (1). Previous studies on bifurcation disease have reported worse outcomes with advanced age (24, 25). Older patients often present with atypical symptoms and non-diagnostic ECG findings, which delay proper diagnosis (2). They also have a higher prevalence of comorbidities (3), and treatment is often complicated by bleeding, renal failure, and neurological impairment (26). Our large dataset reflects the real-world experience of complex PCI using second-generation DESs for bifurcation CAD. In our study, patients with advanced age (≥75 years) presented with more complex CAD (diffuse, calcified, and LMCA) as well as a higher prevalence of hypertension, CKD, prior MI, and previous coronary revascularization. They also had a higher proportion of ACS as a clinical indication for PCI (*p *= 0.094). Nevertheless, the incidence of the primary outcome was comparable between older and younger patients in our study.

Our primary analysis precluded all-cause death and MI to mitigate the effect of age on adverse clinical outcomes. Advanced age was a strong predictor of spontaneous MI in medically managed patients with non-ST-segment elevation ACS (7). Advanced age was also linked to higher all-cause death after PCI (4). Similarly, age as a continuous variable was correlated with the adjusted risk of combined all-cause death and any MI in the current analysis (Figure 3). Thus, all-cause death was a competing event for the primary outcome in our analysis. The Fine-Gray competing risk regression model showed that age ≥75 years was not an independent predictor of the primary outcome (Table 4). Thus, performing complex PCI for bifurcation CAD now seems feasible in older patients. Age-related factors such as postprocedural complications or more frequent underlying comorbidities in older patients can explain the increased risk of all-cause death and any MI after complex bifurcation stenting.

In our multivariable Cox analysis, CKD and LMCA were independent predictors of adverse outcomes regardless of age (Supplementary Table S1), consistent with the results of previous studies (13–15). Clinical outcomes, including cardiac death, were also worse with the two-stent strategy in patients with bifurcation CAD (13, 27). Similarly, the two-stent strategy was associated with a significant risk for the primary outcome in this study (Table 3). Notably, traditional risk factors, such as diabetes and reduced LV systolic function, did not reach statistical significance in the older group, probably due to the limited number of patients. Subgroup analysis showed that the impact of advanced age on clinical outcomes was more obvious in the non-diabetic subgroup (Supplementary Figure S3).

Although PS matching is a technique that attempts to estimate the effect of a treatment or intervention by accounting for the covariates that predict receiving the treatment or intervention, the results of the PS-matched analysis demonstrated the robustness of the multivariable-adjusted analysis. However, this should be interpreted with caution, since the likelihood of being old (≥75 years) is neither treatment nor intervention (Supplementary Table S2).

### 4.1. Limitations

Although our study included a large number of Korean (47.8%) and European (52.2%) patients, it has a few limitations. Using real-world registry data, this retrospective observational study design was associated with an inherent selection bias. It should also be acknowledged that PCI techniques have improved since the initiation of each registry. Moreover, information on the use of intracoronary imaging was not available, making it difficult to reflect the contemporary performance of PCI. The COBIS III registry previously reported 39.8% (1054/2648) of intravascular ultrasound guidance during PCI (13). In addition, the present dataset included only the results of PCI with second-generation DES implantation, not allowing any direct comparison with older patients who were treated with medical therapy alone for bifurcation CAD. Some important patient-centered variables, such as frailty and functional disability in older patients, were not available, providing limited information to clarify high-risk patients who could derive the greatest benefit from complex invasive procedures. Lastly, the BIFURCAT database lacks information on bleeding events, which could be an additional relevant outcome in older patients undergoing complex PCI.

## 5. Conclusion

Among patients with coronary bifurcation stenting, older patients (aged ≥75 years) had an acceptable occurrence of target vessel MI, clinically driven TLR, and ST compared to younger patients (aged <75 years). Increasing age was independently associated with the adjusted risk of combined all-cause death and any MI. CKD and LMCA were independent predictors of adverse cardiac events regardless of age group. Therefore, advanced age alone should not deter physicians from performing complex PCIs for bifurcation CAD.

## Data Availability

The raw data supporting the conclusions of this article will be made available upon request; further enquiries can be directed to the corresponding author.
