# Supplementary material for: Two-Year clinical outcomes after coronary bifurcation stenting in older patients from Korea and Italy
Source: Front Cardiovasc Med. 2023 Mar 23;10:1106594. doi: 10.3389/fcvm.2023.1106594 (PMC10076885; doi:10.3389/fcvm.2023.1106594)

**Supplementary Material**

This appendix was provided by the authors to supply readers with additional information regarding the current study.

1. **Supplemental Tables**

**Table 1.** Predictors of the primary outcome according to age subgroup

**Table 2.** Clinical characteristics before and after propensity score matching

**Table 3.** Incidence and risk of the primary and secondary outcomes at 2 years before and

after propensity score matching.

1. **Supplemental Figures**

**Figure 1.** Flow chart of the study population

**Figure 2**. Distribution of age in the overall study patients

**Figure 3.** Subgroup analysis for the primary outcome

**Table 1.** Predictors of the primary outcome according to age subgroup.

| Variables | Univariable  HR (95% CI) | *p* value | Multivariable  HR (95% CI) | *p* value |
| --- | --- | --- | --- | --- |
| **Age ≥ 75 years** |  |  |  |  |
| Hypertension | 0.97 [0.52–1.83] | 0.934 | 0.94 [0.50–1.80] | 0.860 |
| Diabetes | 0.92 [0.51–1.67] | 0.788 | 0.94 [0.51–1.74] | 0.847 |
| Chronic kidney disease | 1.54 [0.82–2.91] | 0.180 | 1.85 [0.94–3.63] | 0.073 |
| Previous PCI/CABG | 0.90 [0.47–1.72] | 0.754 | 0.77 [0.39–1.49] | 0.432 |
| LV ejection fraction <40% | 1.07 [0.43–2.71] | 0.878 | 1.06 [0.41–2.70] | 0.907 |
| Presentation as acute coronary syndrome | 0.69 [0.40–1.19] | 0.181 | 0.73 [0.42–1.27] | 0.263 |
| LMCA | 2.35 [1.35–4.08] | 0.002 | 2.35 [1.31–4.21] | 0.004 |
| True bifurcation lesion | 1.22 [0.70–2.12] | 0.483 | 1.15 [0.62–2.14] | 0.660 |
| Severe calcification | 0.78 [0.40–1.52] | 0.466 | 0.62 [0.31–1.23] | 0.107 |
| Two-stent strategy | 2.06 [1.15–3.70] | 0.015 | 1.74 [0.89–3.43] | 0.148 |
| **Age < 75 years** |  |  |  |  |
| Hypertension | 1.75 [1.21–2.54] | 0.003 | 1.48 [1.01–2.17] | 0.042 |
| Diabetes | 1.90 [1.36–2.64] | <0.001 | 1.51 [1.07–2.14] | 0.019 |
| Chronic kidney disease | 2.81 [1.79–4.40] | <0.001 | 2.03 [1.25–3.28] | 0.004 |
| Previous PCI/CABG | 1.45 [0.99–2.11] | 0.054 | 1.11 [0.76–1.64] | 0.582 |
| LV ejection fraction <40% | 2.10 [1.21–3.64] | 0.009 | 1.81 [1.03–3.19] | 0.040 |
| Presentation as acute coronary syndrome | 0.88 [0.63–1.23] | 0.466 | 0.93 [0.67–1.31] | 0.693 |
| LMCA | 2.14 [1.54–2.98] | <0.001 | 1.95 [1.38–2.77] | < 0.001 |
| True bifurcation lesion | 1.40 [1.00–1.95] | 0.048 | 1.22 [0.84–1.78] | 0.289 |
| Severe calcification | 1.28 [0.84–1.94] | 0.254 | 0.85 [0.55–1.32] | 0.472 |
| Two-stent strategy | 2.60 [1.84–3.68] | <0.001 | 2.09 [1.42–3.08] | < 0.001 |

CI, confidence interval; CABG, coronary artery bypass graft surgery; HR, hazard ratio; PCI, percutaneous coronary intervention; LV, left ventricle; LMCA, left main coronary artery disease.

**Table 2.** Clinical characteristics before and after propensity score matching.

|  | Overall cohort (n=5,537) | | | |  | Propensity score-matched cohort (n=2,634) | | | |
| --- | --- | --- | --- | --- | --- | --- | --- | --- | --- |
|  | Age < 75  (n=4,122) | Age ≥ 75  (n=1,415) | *P* value | SMD |  | Age < 75  (n=1,317) | Age ≥ 75  (n=1,317) | *P* value | SMD |
| Age (years) | 61.5 ± 8.9 | 80.1 ± 4.0 | < 0.001 |  |  | 63.3 ± 8.3 | 80.1 ± 4.0 | < 0.001 |  |
| Male | 3305 (80.2%) | 923 (65.2%) | < 0.001 | -0.3194 |  | 906 (68.8%) | 875 (66.4%) | 0.212 | -0.0494 |
| Hypertension | 2562 (62.2%) | 1095 (77.4%) | < 0.001 | 0.3621 |  | 1025 (77.8%) | 1018 (77.3%) | 0.779 | -0.0128 |
| Hyperlipidemia | 2026 (49.2%) | 710 (50.2%) | 0.525 | 0.0232 |  | 687 (52.2%) | 668 (50.7%) | 0.483 | -0.0289 |
| Diabetes | 1357 (32.9%) | 477 (33.7%) | 0.609 | 0.0305 |  | 404 (30.7%) | 448 (34.0%) | 0.073 | 0.0706 |
| Current smoker | 1274 (30.9%) | 134 ( 9.5%) | < 0.001 | -0.7460 |  | 119 (9.0%) | 127 (9.6%) | 0.639 | 0.0209 |
| History of MI | 626 (15.2%) | 318 (22.5%) | < 0.001 | 0.1693 |  | 257 (19.5%) | 284 (21.6%) | 0.210 | 0.0488 |
| History of CABG | 96 (2.3%) | 61 (4.3%) | < 0.001 | 0.1004 |  | 53 (4.0%) | 55 (4.2%) | 0.922 | 0.0073 |
| History of PCI | 892 (21.6%) | 352 (24.9%) | 0.013 | 0.0706 |  | 333 (25.3%) | 330 (25.1%) | 0.928 | -0.0053 |
| Chronic kidney disease | 383 (9.3%) | 335 (23.7%) | < 0.001 | 0.3470 |  | 276 (21.0%) | 291 (22.1%) | 0.507 | 0.0265 |
| LV ejection fraction, % | 58.3 ± 9.1 | 56.2 ± 10.5 | < 0.001 | -0.2009 |  | 56.7 ± 9.6 | 56.5 ± 10.2 | 0.626 | -0.0179 |
| Acute coronary syndrome | 2378 (57.7%) | 853 (60.3%) | 0.094 | 0.0473 |  | 761 (57.8%) | 788 (59.8%) | 0.303 | 0.0419 |
| LMCA | 1178 (28.6%) | 469 (33.1%) | 0.001 | 0.1063 |  | 416 (31.6%) | 425 (32.3%) | 0.738 | 0.0146 |
| Severe calcification | 619 (15.0%) | 347 (24.5%) | < 0.001 | 0.2173 |  | 302 (22.9%) | 307 (23.3%) | 0.853 | 0.0089 |
| Diffuse lesion | 1488 (36.1%) | 557 (39.4%) | 0.030 | 0.0762 |  | 487 (37.0%) | 508 (38.6%) | 0.422 | 0.0326 |
| True bifurcation lesion | 1912 (46.4%) | 733 (51.8%) | < 0.001 | 0.1098 |  | 668 (50.7%) | 678 (51.5%) | 0.726 | 0.0152 |

Values are presented as the mean ± standard deviation or n (%). CABG, coronary artery bypass graft surgery; LV, left ventricle; LMCA, left main coronary artery disease; MI, myocardial infarction; PCI, percutaneous coronary intervention.

**Table 3.** Incidence and risk of the primary and secondary outcomes at 2 years before and after propensity score matching.

|  | Overall cohort (n=5,537) | | | |  | Propensity score-matched cohort (n=2,634) | | | |
| --- | --- | --- | --- | --- | --- | --- | --- | --- | --- |
|  | Age < 75  (n=4,122) | Age ≥ 75  (n=1,415) | Unadjusted  HR (95% CI) | Log-rank P |  | Age < 75  (n=1,317) | Age ≥ 75  (n=1,317) | Unadjusted  HR (95% CI) | Log-rank P |
| **Primary outcome** | 141 (4.5%) | 51 (5.7%) | 1.23 (0.89–1.69) | 0.21 |  | 42 (4.5%) | 46 (5.5%) | 1.25 (0.82–1.90) | 0.30 |
| **Secondary outcomes** |  |  |  |  |  |  |  |  |  |
| Target vessel MI | 39 (1.3%) | 15 (1.7%) | 1.31 (0.72–2.38) | 0.38 |  | 16 (1.7%) | 14 (1.8%) | 0.99 (0.48–2.02) | 0.97 |
| Target lesion revascularization | 114 (3.9%) | 36 (4.3%) | 1.11 (0.76–1.62) | 0.58 |  | 37 (4.1%) | 34 (4.3%) | 1.08 (0.68–1.72) | 0.76 |
| Stent thrombosis | 45 (1.3%) | 19 (1.8%) | 1.35 (0.79–2.30) | 0.28 |  | 14 (1.4%) | 16 (1.7%) | 1.25 (0.61–2.56) | 0.55 |
| Any MI | 86 (2.7%) | 48 (4.9%) | 1.84 (1.29–2.61) | 0.001 |  | 27 (3.1%) | 44 (4.9%) | 1.56 (0.98–2.47) | 0.06 |
| All-cause death + any MI | 163 (5.2%) | 136 (14.1%) | 2.80 (2.23–3.52) | <0.001 |  | 63 (6.5%) | 117 (13.1%) | 2.08 (1.53–2.83) | <0.001 |

Values are presented as numbers (an estimate of the cumulative incidence of events over time). CI, confidence interval; HR, hazard ratio; MI, myocardial infarction.

**Figure 1.** Flow chart of the study population.


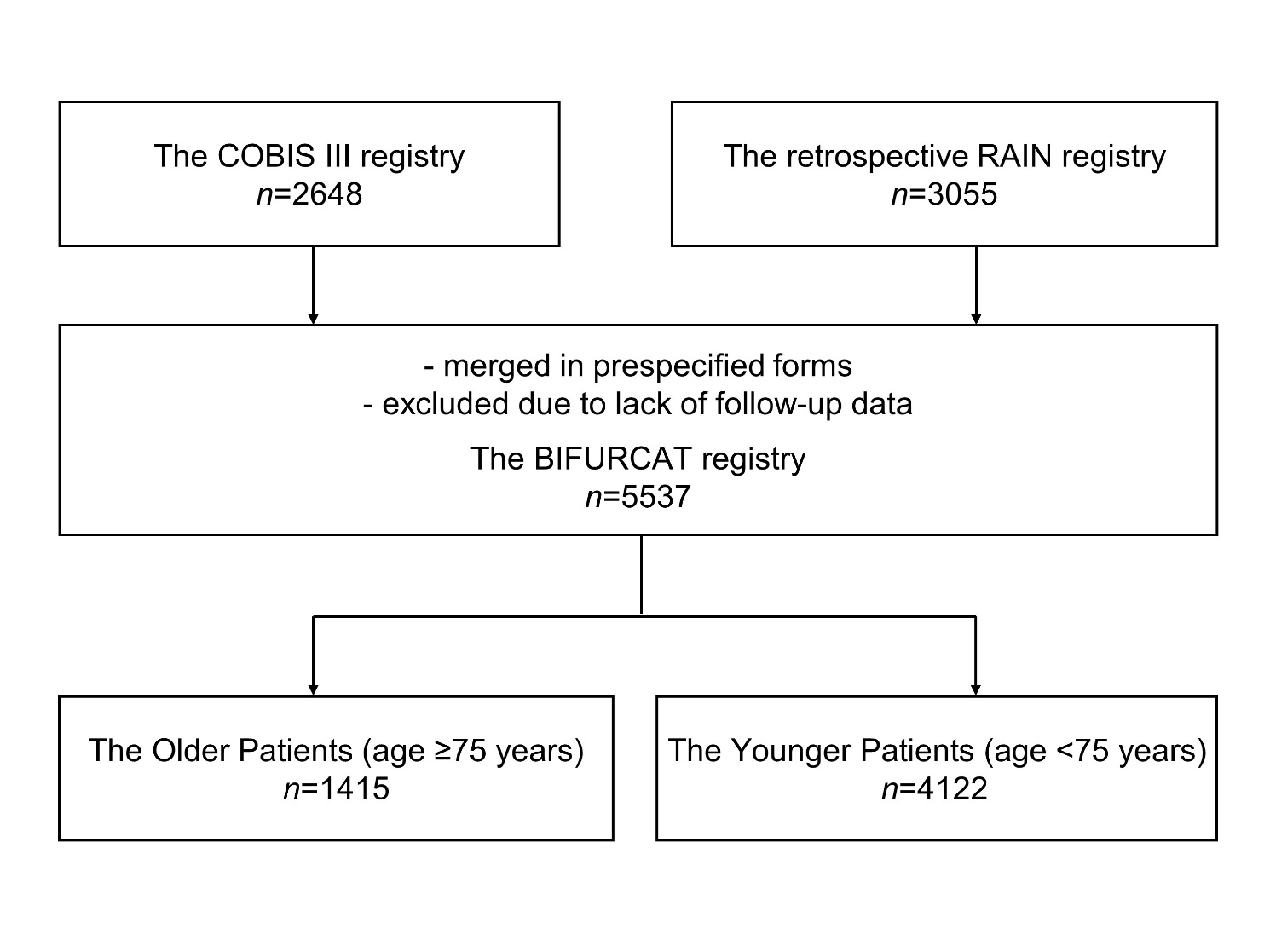


**Figure 2**. Distribution of age in the overall study patients.


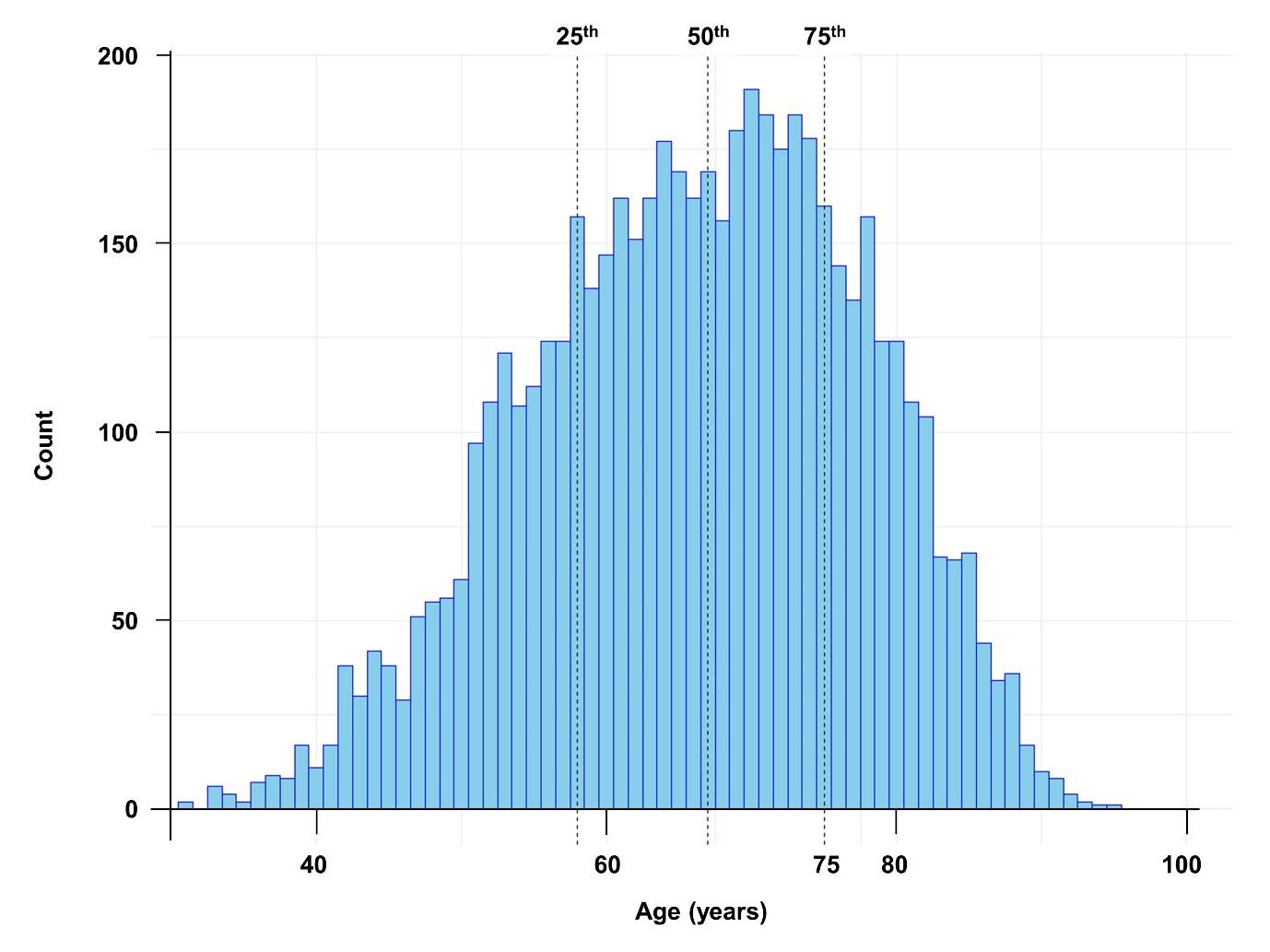


**Figure 3.** Subgroup analysis for the primary outcome.


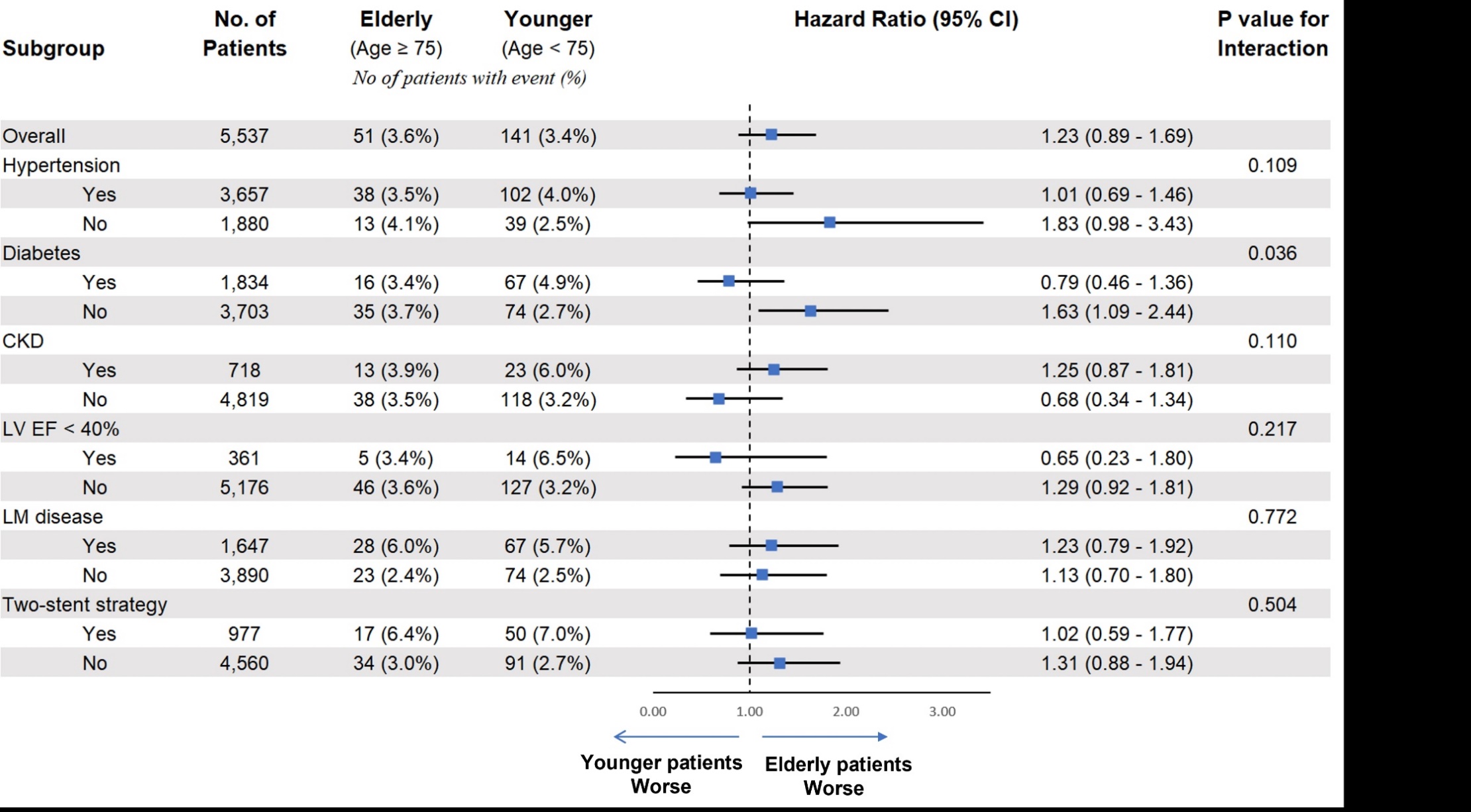

Supplement: Supplementary file 1 [file Table2.docx]
